# Supplementary material for: A Neutropenic Diet in Haemato-Oncological Patients Receiving High-Dose Therapy and Hematopoietic Stem Cell Transplantation: A Systematic Review
Source: Nutrients. 2025 Feb 21;17(5):768. doi: 10.3390/nu17050768 (PMC11901642; doi:10.3390/nu17050768)
Supplement: Supplementary file 1 [file nutrients-17-00768-s001.zip › nutrients-3470167-supplementary.pdf]

nutrients

MDPI

Systematic Review

A Neutropenic Diet in Haemato-Oncological Patients Receiving High-dose Therapy and Hematopoietic Stem Cell Transplantation: A Systematic Review

Luise Jahns [1,2,*], Jutta Hübner [2], Christina Mensger [2] and Viktoria Mathies [2]

[1] Institute of Agricultural and Nutritional Sciences, Martin Luther University, 06120 Halle, Germany
[2] Department of Hematology and Internal Oncology, University Hospital Jena, 07747 Jena, Germany; jutta.huebner@med.uni-jena.de (J.H.); christina.mensger@med.uni-jena.de (C.M.); viktoria.mathies@med.uni-jena.de (V.M.)
* Correspondence: luise.jahns@student.uni-halle.de

Supplementary Materials

Studies	Sonbol (2015) n=4	Van Dalen (2016) n=3	Ball (2019) n=5	Sonbol (2019) n=6	Ramamoorthy (2020) n=11	Ma (2022) n=6	Matteucci (2023) n=12
Sonbol (2015)							✓
van Dalen (2016)					✓		
Ball (2019)					✓		✓
Sonbol (2019)					✓		
Ramamoorthy (2020)							
Ma (2022)							✓
Matteucci (2023)							
van Tiel (2007)	✓	✓	✓	✓	✓	✓	
Gardner (2008)	✓	✓	✓	✓	✓	✓	
Lassiter (2015)			✓	✓	✓	✓	
Radhakrishnan (2022)							
Stella (2023)							
additional studies included	1 retrospective 1 paediatric study	1 paediatric study	2 paediatric studies	1 retrospektive 2 paediatric studies	1 retropective 3 paediatric 1 descriptive	1 retrospektive 2 paediatric	9 Cross-sectional and case-control studies, literature studies
SR/MA							
RCTs							

Figure S1: Literature overview: an overview of SRs relevant to the topic and the publications included in each review

 https://doi.org/10.3390/nu17050768

Table S1: PRISMA checklist

Section and Topic	Item #	Checklist item	Location where item is reported
TITLE			
Title	1	Identify the report as a systematic review.	title
ABSTRACT			
Abstract	2	See the PRISMA 2020 for Abstracts checklist.	table S2
INTRODUCTION			
Rationale	3	Describe the rationale for the review in the context of existing knowledge.	Introduction
Objectives	4	Provide an explicit statement of the objective(s) or question(s) the review addresses.	last paragraph of introduction
METHODS			
Eligibility criteria	5	Specify the inclusion and exclusion criteria for the review and how studies were grouped for the syntheses.	section 2.1 and table 1
Information sources	6	Specify all databases, registers, websites, organisations, reference lists and other sources searched or consulted to identify studies. Specify the date when each source was last searched or consulted.	section 2.2 and supplementary material (table S3 – S6)
Search strategy	7	Present the full search strategies for all databases, registers and websites, including any filters and limits used.	supplementary material (table S3 – S6)
Selection process	8	Specify the methods used to decide whether a study met the inclusion criteria of the review, including how many reviewers screened each record and each report retrieved, whether they worked independently, and if applicable, details of automation tools used in the process.	section 2.1 and 2.2
Data collection process	9	Specify the methods used to collect data from reports, including how many reviewers collected data from each report, whether they worked independently, any processes for obtaining or confirming data from study investigators, and if applicable, details of automation tools used in the process.	section 2.3
Data items	10a	List and define all outcomes for which data were sought. Specify whether all results that were compatible with each outcome domain in each study were sought (e.g. for all measures, time points, analyses), and if not, the methods used to decide which results to collect.	table 1, table 2
	10b	List and define all other variables for which data were sought (e.g. participant and intervention characteristics, funding sources). Describe any assumptions made about any missing or unclear information.	table 2
Study risk of bias assessment	11	Specify the methods used to assess risk of bias in the included studies, including details of the tool(s) used, how many reviewers assessed each study and whether they worked independently, and if applicable, details of automation tools used in the process.	section 2.4
Effect measures	12	Specify for each outcome the effect measure(s) (e.g. risk ratio, mean difference) used in the synthesis or presentation of results.	not applicable
Synthesis methods	13a	Describe the processes used to decide which studies were eligible for each synthesis (e.g. tabulating the study intervention characteristics and comparing against the planned groups for each synthesis (item #5)).	section 2.2, information about eligible outcomes per study see table 2
	13b	Describe any methods required to prepare the data for presentation or synthesis, such as handling of missing summary statistics, or data conversions.	not applicable (narrative synthesis)
	13c	Describe any methods used to tabulate or visually display results of individual studies and syntheses.	not applicable
	13d	Describe any methods used to synthesize results and provide a rationale for the choice(s). If meta-analysis was performed, describe the model(s), method(s) to identify the presence and extent of statistical heterogeneity, and software package(s) used.	not applicable

Section and Topic	Item #	Checklist item	Location where item is reported
	13e	Describe any methods used to explore possible causes of heterogeneity among study results (e.g. subgroup analysis, meta-regression).	not applicable
	13f	Describe any sensitivity analyses conducted to assess robustness of the synthesized results.	not done, but risk of bias assessment was done
Reporting bias assessment	14	Describe any methods used to assess risk of bias due to missing results in a synthesis (arising from reporting biases).	not done
Certainty assessment	15	Describe any methods used to assess certainty (or confidence) in the body of evidence for an outcome.	not done, but risk of bias assessment was done
RESULTS			
Study selection	16a	Describe the results of the search and selection process, from the number of records identified in the search to the number of studies included in the review, ideally using a flow diagram.	figure 1 and section 3.1
	16b	Cite studies that might appear to meet the inclusion criteria, but which were excluded, and explain why they were excluded.	section 3.2
Study characteristics	17	Cite each included study and present its characteristics.	section 3.1, table 2
Risk of bias in studies	18	Present assessments of risk of bias for each included study.	Figure 2
Results of individual studies	19	For all outcomes, present, for each study: (a) summary statistics for each group (where appropriate) and (b) an effect estimate and its precision (e.g. confidence/credible interval), ideally using structured tables or plots.	table 2
Results of syntheses	20a	For each synthesis, briefly summarise the characteristics and risk of bias among contributing studies.	figure 2
	20b	Present results of all statistical syntheses conducted. If meta-analysis was done, present for each the summary estimate and its precision (e.g. confidence/credible interval) and measures of statistical heterogeneity. If comparing groups, describe the direction of the effect.	not done (narrative analysis)
	20c	Present results of all investigations of possible causes of heterogeneity among study results.	not done (narrative analysis)
	20d	Present results of all sensitivity analyses conducted to assess the robustness of the synthesized results.	not done (narrative analysis)
Reporting biases	21	Present assessments of risk of bias due to missing results (arising from reporting biases) for each synthesis assessed.	not done
Certainty of evidence	22	Present assessments of certainty (or confidence) in the body of evidence for each outcome assessed.	not done, but risk of bias assessment was done
DISCUSSION			
Discussion	23a	Provide a general interpretation of the results in the context of other evidence.	section 4
	23b	Discuss any limitations of the evidence included in the review.	section 4.1 – 4.6
	23c	Discuss any limitations of the review processes used.	section 4.8
	23d	Discuss implications of the results for practice, policy, and future research.	section 4.9
OTHER INFORMATION			
Registration and protocol	24a	Provide registration information for the review, including register name and registration number, or state that the review was not registered.	section 2, first paragraph
	24b	Indicate where the review protocol can be accessed, or state that a protocol was not prepared.	protocol was not prepared but registered with OSF

Section and Topic	Item #	Checklist item	Location where item is reported
	24c	Describe and explain any amendments to information provided at registration or in the protocol.	protocol was not prepared but registered with OSF
Support	25	Describe sources of financial or non-financial support for the review, and the role of the funders or sponsors in the review.	no funding
Competing interests	26	Declare any competing interests of review authors.	no conflicts of interest
Availability of data, code and other materials	27	Report which of the following are publicly available and where they can be found: template data collection forms; data extracted from included studies; data used for all analyses; analytic code; any other materials used in the review.	Data availability statement

From: Page MJ, McKenzie JE, Bossuyt PM, Boutron I, Hoffmann TC, Mulrow CD, et al. The PRISMA 2020 statement: an updated guideline for reporting systematic reviews. BMJ 2021;372:n71. doi: 10.1136/bmj.n71. This work is licensed under CC BY 4.0. To view a copy of this license, visit https://creativecommons.org/licenses/by/4.0/

Table S2: PRISMA abstract checklist

Section and Topic	Item #	Checklist item	Reported (Yes/No)
TITLE			
Title	1	Identify the report as a systematic review.	yes
BACKGROUND			
Objectives	2	Provide an explicit statement of the main objective(s) or question(s) the review addresses.	yes
METHODS			
Eligibility criteria	3	Specify the inclusion and exclusion criteria for the review.	yes
Information sources	4	Specify the information sources (e.g. databases, registers) used to identify studies and the date when each was last searched.	yes
Risk of bias	5	Specify the methods used to assess risk of bias in the included studies.	no
Synthesis of results	6	Specify the methods used to present and synthesise results.	not applicable
RESULTS			
Included studies	7	Give the total number of included studies and participants and summarise relevant characteristics of studies.	yes
Synthesis of results	8	Present results for main outcomes, preferably indicating the number of included studies and participants for each. If meta-analysis was done, report the summary estimate and confidence/credible interval. If comparing groups, indicate the direction of the effect (i.e. which group is favoured).	yes
DISCUSSION			
Limitations of evidence	9	Provide a brief summary of the limitations of the evidence included in the review (e.g. study risk of bias, inconsistency and imprecision).	no
Interpretation	10	Provide a general interpretation of the results and important implications.	yes
OTHER			
Funding	11	Specify the primary source of funding for the review.	no funding
Registration	12	Provide the register name and registration number.	no

From: Page MJ, McKenzie JE, Bossuyt PM, Boutron I, Hoffmann TC, Mulrow CD, et al. The PRISMA 2020 statement: an updated guideline for reporting systematic reviews. BMJ 2021;372:n71. doi: 10.1136/bmj.n71. This work is licensed under CC BY 4.0. To view a copy of this license, visit https://creativecommons.org/licenses/by/4.0/

Search string

Table S3: MEDLINE (Ovid) search string

Line	Search
1	Stem Cell Transplantation/ OR stem cell transplant*.mp. OR Hematopoietic Stem Cell Transplantation/ OR stem cell support.mp. OR Bone Marrow Transplantation/ OR bone marrow transplant*.mp. OR exp febrile neutropenia/ or (neutropen* adj20 (neoplasm* or cancer* or tumo?r* or malignan* or oncolog* or carcinoma* or leuk?emia or lymphoma* or sarcom* or myelom* or chemotherap*)).tw. or ((high dose or intensive or myelosuppressive or myelotoxic or induction) adj1 (chemotherap* or therap* or treatment)).tw.
2	exp nutrition therapy/ OR *feeding methods/ or exp enteral nutrition/ or exp parenteral nutrition/ OR exp Dietary Supplements/ OR exp immunonutrition diet/ OR nutrition*.mp. OR diet*.mp. OR nourish*.mp. OR enteral.mp OR parenteral.mp. OR ((neutropenic* or low-bacteria*) adj6 diet*).mp.
3	1 AND 2
4	limit 3 to english or limit 3 to german
5	(4 and humans/) or (4 not animals/)
6	(((comprehensive* or integrative or systematic*) adj3 (bibliographic* or review* or literature)) or (meta-analy* or metaanaly* or "research synthesis" or ((information or data) adj3 synthesis) or (data adj2 extract*))).ti,ab. or (cinahl or (cochrane adj3 trial*) or embase or medline or psyclit or (psycinfo not "psycinfo database") or pubmed or scopus or "sociological abstracts" or "web of science").ab. or ("cochrane database of systematic reviews" or evidence report technology assessment or evidence report technology assessment summary).jn. or Evidence Report: Technology Assessment*.jn. or ((review adj5 (rationale or evidence)).ti,ab. and review.pt.) or meta-analysis as topic/ or Meta-Analysis.pt.
7	"clinical trial".pt. or "clinical trial, phase i".pt. or "clinical trial, phase ii".pt. or clinical trial, phase iii.pt. or clinical trial, phase iv.pt. or controlled clinical trial.pt. or "multicenter study".pt. or "randomized controlled trial".pt. or double-blind method/ or clinical trials as topic/ or clinical trials, phase i as topic/ or clinical trials, phase ii as topic/ or clinical trials, phase iii as topic/ or clinical trials, phase iv as topic/ or controlled clinical trials as topic/ or randomized controlled trials as topic/ or early termination of clinical trials as topic/ or multicenter studies as topic/ or ((analyi?ed adj7 trial*) or (controlled adj3 trial*) or (clinical adj2 trial*) or ((single or doubl* or tripl* or treb*) and (blind* or mask*))).ti,ab,kw. or ("4 arm" or "four arm").ti,ab,kw or randomi?ed.ti,ab,kw.
8	5 AND (6 OR 7)

Table S4: Embase (Ovid) search string

Line	Search
1	Stem Cell Transplantation/ OR Hematopoietic Stem Cell Transplantation/ OR Bone Marrow Transplantation/ OR exp febrile neutropenia/ OR stem cell transplant*.mp. OR stem cell support.mp. OR bone marrow transplant*.mp. OR (neutropen* adj20 (neoplasm* OR cancer* OR tumo?r* OR malignan* OR oncolog* OR carcinoma* OR leuk?emia OR lymphoma* OR sarcom* OR myelom* OR chemotherap*)).tw. OR (("high dose" OR intensive OR myelosuppressive OR myelotoxic OR induction) adj1 (chemotherap* OR therap* OR treatment)).tw.
2	diet therapy/ OR enteric feeding/ OR parenteral nutrition/ OR total parenteral nutrition/ OR dietary supplement/ OR immunonutrition/ OR nutrition*.tw. OR diet*.tw. OR nourish*.tw. OR enteral.tw. OR parenteral.tw. OR ((neutropenic* or low-bacteria*) adj6 diet*).tw.
3	1 AND 2
4	limit 3 to english or limit 3 to german
5	(4 and humans/) OR (4 not animals/)

6	(((comprehensive* or integrative or systematic*) adj3 (bibliographic* or review* or literature)) or (meta-analy* or metaanaly* or "research synthesis" or ((information or data) adj3 synthesis) or (data adj2 extract*))).ti,ab. or (cinahl or (cochrane adj3 trial*) or embase or medline or psyclit or (psycinfo not "psycinfo database") or pubmed or scopus or "sociological abstracts" or "web of science").ab. or ("cochrane database of systematic reviews" or evidence report technology assessment or evidence report technology assessment summary).jn. or Evidence Report: Technology Assessment*.jn. or (exp Meta Analysis/ or ((data extraction.ab. or selection criteria.ab.) and review.pt.))
7	crossover procedure/ or double blind procedure/ or randomized controlled trial/ or single blind procedure/ or (random$ or factorial$ or crossover$ or (cross adj1 over$) or placebo$ or (doubl$ adj1 blind$) or (singl$ adj1 blind$) or assign$ or allocat$ or volunteer$).ti,ab,de.
8	5 AND (6 OR 7)

Table S5: CINAHL (EBSCO) search string

**Line**	**Search**
S1	(MH "Hematopoietic Stem Cell Transplantation") OR (MH "Bone Marrow Transplantation+") OR (MH "Neutropenia+") OR<br>"stem cell transplant*" OR "stem cell support" OR "bone marrow transplant*" OR ((TI neutropen* OR AB neutropen*) N20 ((TI neoplasm* OR AB neoplasm*) OR (TI cancer* OR AB cancer*) OR (TI tumo#r* OR AB tumo#r*) OR (TI malignan* OR AB malignan*) OR (TI oncolog* OR AB oncolog*) OR (TI carcinoma* OR AB carcinoma*) OR (TI leuk#emia OR AB leuk#emia) OR (TI lymphoma* OR AB lymphoma*) OR (TI sarcom* OR AB sarcom*) OR (TI myelom* OR AB myelom*) OR (TI chemotherap* OR AB chemotherap*))) OR (((TI "high dose" OR AB "high dose") OR (TI intensive OR AB intensive) OR (TI myelosuppressive OR AB myelosuppressive) OR (TI myelotoxic OR AB myelotoxic) OR (TI induction OR AB induction)) N1 ((TI chemotherap* OR AB chemotherap*) OR (TI therap* OR AB therap*) OR (TI treatment OR AB treatment)))
S2	MM "Diet Therapy") or (MM "Nutrition") or (MH "Immunonutrition Diet") or (MM "Nutritional Support") or (MM "Dietary Supplementation") or (MM "Enteral Nutrition") or (MM "Home Nutritional Support") or (MM "Parenteral Nutrition") OR (TI nutrition* OR AB nutrition*) OR (TI diet* OR AB diet*) OR (TI nourish* OR TI nourish*) OR (TI enteral OR AB enteral) OR (TI parenteral OR AB parenteral) OR ((TI neutropenic* OR AB neutropenic*) OR ((TI low-bacteria* OR AB low-bacteria*) N6 (TI diet* OR AB diet*))
S3	LA German OR LA English
S4	S1 AND S2 AND S3
S5	(TI (systematic* n3 review*)) or (AB (systematic* n3 review*)) or (TI (systematic* n3 bibliographic*)) or (AB (systematic* n3 bibliographic*)) or (TI (systematic* n3 literature)) or (AB (systematic* n3 literature)) or (TI (comprehensive* n3 literature)) or (AB (comprehensive* n3 literature)) or (TI (comprehensive* n3 bibliographic*)) or (AB (comprehensive* n3 bibliographic*)) or (TI (integrative n3 review)) or (AB (integrative n3 review)) or (JN "Cochrane Database of Systematic Reviews") or (TI (information n2 synthesis)) or (TI (data n2 synthesis)) or (AB (information n2 synthesis)) or (AB (data n2 synthesis)) or (TI (data n2 extract*)) or (AB (data n2 extract*)) or (TI (medline or pubmed or psyclit or cinahl or (psycinfo not "psycinfo database") or "web of science" or scopus or embase)) or (AB (medline or pubmed or psyclit or cinahl or (psycinfo not "psycinfo database") or "web of science" or scopus or embase)) or (MH "Systematic Review") or (MH "Meta Analysis") or (TI (meta-analy* or metaanaly*)) or (AB (meta-analy* or metaanaly*))
S6	(MH "Clinical Trials+") or PT Clinical trial or TX clinic* n1 trial* or TX ((singl* n1 blind*) or (singl* n1 mask*)) or TX ((doubl* n1 blind*) or (doubl* n1 mask*)) or TX ((tripl* n1 blind*) or (tripl* n1 mask*)) or TX ((trebl* n1 blind*) or (trebl* n1 mask*)) or TX randomi* control* trial* or (MH "Random Assignment") or TX random* allocat* or TX placebo* or MH "Placebos") or MH "Quantitative Studies") or TX allocat* random*
S7	S4 AND (S5 OR S6)

Table S6: Cochrane search string

Line	Search
#1	[mh ^"Stem Cell Transplantation"] OR ("stem cell" NEXT transplant*):ti,ab,kw OR [mh ^"Hematopoietic Stem Cell Transplantation"] OR "stem cell support":ti,ab,kw OR [mh ^"Bone Marrow Transplantation"] OR ("bone marrow" NEXT transplant*):ti,ab,kw OR [mh "febrile neutropenia"] OR (neutropen*:ti,ab NEAR/20 (neoplasm*:ti,ab OR cancer*:ti,ab OR tumo?r*:ti,ab OR malignan*:ti,ab OR oncolog*:ti,ab OR carcinoma*:ti,ab OR leuk?emia:ti,ab OR lymphoma*:ti,ab OR sarcom*:ti,ab OR myelom*:ti,ab OR chemotherap*:ti,ab)) OR (("high dose":ti,ab OR intensive:ti,ab OR myelosuppressive:ti,ab OR myelotoxic:ti,ab OR induction:ti,ab) NEAR/1 (chemotherap*:ti,ab OR therap*:ti,ab OR treatment:ti,ab))
#2	[mh "nutrition therapy"] OR [mh ^"feeding methods"] OR [mh "enteral nutrition"] OR [mh "parenteral nutrition"] OR [mh "Dietary Supplements"] OR [mh "immunonutrition diet"] OR nutrition*:ti,ab,kw OR diet*:ti,ab,kw OR nourish*:ti,ab,kw OR enteral:ti,ab,kw OR parenteral:ti,ab,kw OR ((neutropenic*:ti,ab,kw OR low-bacteria*:ti,ab,kw) NEAR/6 diet*:ti,ab,kw)
#3	#1 AND #2

Table S7. Excluded reviews and studies.

References	Year	Title	Reason for exclusion
DeMille et al. [46]	2006	The effect of the neutropenic diet in the outpatient setting: a pilot study	Publication type: descriptive pilot study
Moody et al. [47]	2006	Feasibility and safety of a pilot randomized trial of infection rate: neutropenic diet versus standard food safety guidelines	Paediatric oncology patients
Tarr et al. [48]	2009	Evidence-based standards for a non-neutropenic diet in an adult inpatient oncology setting	No full text available
Jubelirer [49]	2011	The benefit of the neutropenic diet: Fact or fiction?	Publication type: narrative review
Foster [50]	2014	Reevaluating the Neutropenic Diet: Time to Change	Publication type: narrative review
Sonbol et al. [45]	2015	The Effect of a Neutropenic Diet on Infection and Mortality Rates in Cancer Patients: A Meta-Analysis	Publication type: SR
Van Dalen et al. [7]	2016	Low bacterial diet versus control diet to prevent infection in cancer patients treated with chemotherapy causing episodes of neutropenia	Publication type: SR
Wolfe et al. [15]	2018	Things We Do For No Reason: Neutropenic Diet	Publication type: narrative review
Moody [51]	2019	Neutropenic Dietary Restrictions for Hematopoietic Stem Cell Patients: Time for a Change	Publication type: narrative review
Ball et al. [17]	2019	Effect of Neutropenic Diet on Infection Rates in Cancer Patients with Neutropenia: A Meta-analysis of Randomized Controlled Trials	Publication type: SR
Sonbol et al. [8]	2019	Neutropenic diets to prevent cancer infections: updated systematic review and meta-analysis	Publication type: SR
Ramamoorthy et al. [27]	2020	Lack of Efficacy of the Neutropenic Diet in Decreasing Infections among Cancer Patients: A Systematic Review	Publication type: SR
Ma et al. [26]	2022	Neutropenic Diet Cannot Reduce the Risk of Infection and Mortality in Oncology Patients With Neutropenia	Publication type: SR
Matteucci et al. [14]	2023	Low-bacterial diet in cancer patients: a systematic review	Publication type: SR

Table S8: Risk of Bias of included studies.

Reference	Study type	Standardized rating of risk of bias	Additional comments on methodology
Van Tiel et al. (2007) [31]	RCT	RoB 2.0: high risk	PRO: use of multivariate analysis of variance to correct baseline differences<br><br>CONTRA: small sample size (n = 20), primary haematological disease differed between groups, definition of infection is inappropriate, no study protocol, open label study, single centre study
Gardner et al. (2008) [32]	RCT	RoB 2.0: some concerns	PRO: large sample size (n = 153), no lost to follow up or discontinued intervention, concealed randomisation, power analysis, no baseline differences<br><br>CONTRA: no study protocol, open label study, single centre
Lassiter et al. (2015) [33]	RCT	RoB 2.0: some concerns	PRO: Balanced baseline characteristics, randomisation process with low bias risk<br><br>CONTRA: small sample size (n = 46), inconsistent reporting: figure 1 and text are contradictory, per protocol analysis, open label study, no study protocol, presentation of PG-SGA-Scores as graph only, single centre study
Radhakrishnan et al. (2022) [24]	RCT	RoB 2.0: some concerns	PRO: large sample (n = 200), balanced baseline characteristics, concealed allocation, statistical power analysis<br><br>CONTRA: open label study, single centre study
Stella et al. (2023) [25]	RCT	RoB 2.0: high risk	PRO: large sample (n = 222), balanced baseline characteristics, multicentre study<br><br>CONTRA: no details on randomisation process, open label study, per protocol analysis *(different as reported),* inconsistent reporting: figure 1 and text are contradictory, study protocol not possible to retrieve (despite reported registration), endpoints and assessment methods are not defined, no results for overall survival reported, QoL was not defined as endpoint at study start (selective outcome reporting)

Abbreviations: RoB – risk of bias; PG-SGA - Patient-Generated Subjective Global Assessment, QoL – Quality of life
